# Personal Service Triumphant

**Published:** 1917-12-22

**Authors:** 


### PERSONAL SERVICE TRIUMPHANT.

By the time this number of The Hospital is being read Christmas Eve will probably have arrived. Anyway our first duty and privilege is to wish all hospital workers throughout the United Kingdom and abroad, including the many new friends who have joined the ranks in this capacity owing to the war, a Joyous Christmas warmed by personal service, yielding abundant fruit, in individual inspiration and blessedness. The actual conditions of war-time as they have affected the voluntary and other hospitals throughout the country, the Empire, and abroad, will this year raise personal service to an even higher standard, seeing that the realisation by the patients of a worthy, inspiriting, uplifting, joyous Christmas Day in 1917 must depend upon the spirit of the workers, and their whole-hearted, self-denying, and best efforts to make everybody remember the day and rejoice over it.

Christmas produces memories for us all in proportion to the length of years during which each one has had the privilege of ministering to the welfare of the sick and suffering. Last week, thanks to many correspondents, we were able to give an idea of how Christmas Day will be spent this year in representative institutions of many types. The perusal of this article (pages 235-6) stirred up many treasured memories of Christmas Day in the voluntary hospitals, where the Editor has had the privilege of taking an active part for continuous years. In one such institution it recalls the memory of what our hospitals in this country were, at Christmas-time fifty years ago. In those days hospital conditions offered scant encouragement for the exhibition of Christmas joy. Their chilling atmosphere was keenly felt by ardent workers, and resulted in changes which secured the recognition of the claims of the inmates of our hospitals, to have the means provided for them to spend Christmas Day in joyous thankfulness, amidst congenial surroundings. The fruits of this improved atmosphere and outlook for the patients, and its recognised value, are demonstrated by the article in question. It affords evidence that the spirit which secured the individual recognition of every patient in a hospital, and was made manifest by a suitable gift given to each so that the surprise packet might be discovered when the patient awoke on Christmas morning, has endured for forty-eight consecutive years. It further demonstrates that for decades of years and now, the seamen patients could enjoy a Christmas as like the old days of the Yule Log as it is possible. These two happenings carry us back to a time preceding the adequate and full training of nurses for their responsible duties. All through the decades the adoption of this inspiriting change has extended and grown, until to-day there is not a British hospital worthy of the name, where Christmas is not celebrated, and Christmas Day is not capable of yielding pleasant memories for the patients, for the rest of their days.

We have spoken on a previous occasion of the inspiring atmosphere of every hospital, receiving station, outpost, and camp connected with our armies, the men of which have fought with such magnificent courage and ennobling results throughout the whole field of operations, in connection with the present terrible war. This atmosphere will surely mingle with that of the hospitals in the Homeland on Christmas Day 1917, and so at least on this one day, wherever hospital workers are rendering personal service, there an identical spirit of inspiration should uplift every one of them, and give them cause to thank God and take courage. Then with grateful hearts they will renew their work, and face, whatever the future may have in store, with thankfulness and hope.

Difficulties are made for the wise to overcome, and we may surely claim that the heartiness of our Christmas greetings to all our friends and fellow-workers can never have attained a higher or fuller import than Christmas Day 1917 will carry with it for everybody. Workers everywhere will welcome the news that one outcome of the article, "A War-time Christmas in Hospitals," in The Hospital of December 8, page 196, has been an intimation that a number "of cold hearts existing in human bodies," to which reference was made, have been warmed and changed. Very many or, as we hope, all of them have decided, to join hands with the army of workers, who have so gratefully rendered personal service to hospital patients in the past, so that a memorable Christmas for everybody in hospital on December 25, 1917, is assured.

